# Experiences and Perceptions of Clinical and Graduate Medical Students Regarding AI in Syria: Cross-Sectional Study

**DOI:** 10.2196/84942

**Published:** 2026-05-19

**Authors:** Jouni Aljoudeh, Abdulrahman Al Balkhi, Yahia Ranjous, Abdulrahman Shbani, Danial Takieddin, Mohammad Bashar Izzat

**Affiliations:** 1 Department of Otolaryngology, Faculty of Medicine, Damascus University, Damascus, Syrian Arab Republic; 2 Gastroenterology Department, Police Hospital, Harasta, Syrian Arab Republic; 3 Faculty of Medicine, Damascus University, Damascus, Syrian Arab Republic; 4 Department of Surgery, Faculty of Medicine, Damascus University, Damascus, Syrian Arab Republic; 5 Faculty of Medicine, Tartous University, Tartous city, Syrian Arab Republic, 963 934853309

**Keywords:** artificial intelligence, AI, ChatGPT, medical education, cross-sectional, Syria

## Abstract

**Background:**

Artificial intelligence (AI) tools have revolutionized various aspects of education and health care in recent years. Their influence extends across multiple domains of medical education, from traditional learning to research and foreign language acquisition.

**Objective:**

This study aims to evaluate the experiences and perceptions of AI tools usage in a low-resource setting and identify the factors influencing their adoption.

**Methods:**

A cross-sectional study was conducted to evaluate the experiences with AI tools and perceptions regarding their future applications in education and health care among medical students in Syria. The sample was equally divided between clinical-year students and graduates. Chi-square tests analyzed differences based on demographics and experience, while Mann-Whitney *U* tests compared group perceptions of AI’s future role. Factors studied included academic year, gender, German language learning, computer access, and research experience.

**Results:**

Among 400 participants, AI tools were widely used for study preparation (228/400, 57% of participants), assignments (160/400, 40% of participants), and research. Clinical students used AI more than graduates for examination preparation (*P*<.001), creating cases (*P*=.03), and writing tasks (*P*<.001). Males used AI more for research (*P*=.004) or anatomy (*P*=.02); German learners relied on AI for language tasks. Despite 76% (304/400) of students believing AI would enhance residency training and 71.8% (287/400) of students supporting institutional policies, only 25.5% (102/400) of students expected career benefits. Ethical concerns were higher among females and researchers.

**Conclusions:**

This study highlights the increasing reliance on AI tools among medical students and graduates for academic and clinical purposes. The highest usage was reported in study preparation, writing tasks, and clinical simulations. Significant differences in AI usage were observed based on academic level, gender, access to technology, and research experience. While perceptions were largely positive, concerns remained around ethical use, potential job displacement, and diminished human interaction in medicine. These findings underscore the importance of developing institutional policies to guide the ethical and effective integration of AI in medical education.

## Introduction

Artificial intelligence (AI) refers to modern systems that use self-learning algorithms to perform tasks and generate responses that mimic human intelligence [1]. Despite its early beginnings, the surge in its popularity and success was in 2022 when OpenAI launched ChatGPT. Within just 5 days, the platform gained one million users, making it the second-fastest application in history to reach this milestone [2,3].

In the medical domain, AI has proved itself as a valuable tool for both education and health care. For students, AI assists in editing texts, generating medical case scenarios, summarizing complex topics, and simplifying difficult information, which saves time and enhances efficiency [4,5]. In health care, AI has proved itself as a valuable tool for medical practitioners. It has passed the USMLEs (United States Medical Licensing Examinations) and proved its efficiency in disease diagnosis across multiple specialties. Its performance is particularly notable in specialties that rely on image analysis, such as radiology and pathology [6-10]. However, these advancements are not without doubts and concerns. The rapid growth of AI has sparked debates over ethical issues, job displacement in health care, and other challenges [11,12].

The integration of AI tools in medical education is crucial, as this new and fast-growing technology will shape the future of health care, despite its absence from traditional medical curricula. The World Medical Association has endorsed AI integration into medical education; however, its implementation is still not sufficient worldwide [13,14]. This gap presents a notable challenge in low-resource areas, where limited access to computers, reliable electricity, and internet connectivity further hinders adoption [15].

It is essential to understand how medical students engage with AI at different stages of their medical training. Clinical-year students (fourth and fifth years) and preresidency students (graduates and near-graduates) differ in both academic needs and clinical responsibilities. For instance, clinical students often rely on AI to prepare for examinations and explain complex information, whereas preresidency students tend to use AI for residency preparation and clinical decision-making. Comparing these groups provides valuable insights into whether AI experiences and perceptions evolve with advancing training and clinical practice.

Syria has endured a devastating war since 2011, resulting in millions of refugees both inside and outside the country [16]. The conflict has huge impacts on all aspects of life, including health care and medical education. Over half of medical facilities were destroyed or damaged during the war [17-19]. Moreover, the concurrent economic collapse and infrastructure devastation have created severe poverty and shortages in necessities, including food and electricity, among others [20]. One notable consequence is that most medical students now intend to immigrate after graduation, with Germany emerging as the primary destination for Syrian doctors [21-23]. These factors have significantly impacted medical education and access to knowledge.

Most existing literature originates from well-resourced settings, leaving limited knowledge about how AI is experienced and perceived in low recourse, conflict-affected contexts. In such settings, limited access to computers, electricity outages, poor internet connection, and lack of institutional support can hinder the effective use of AI. At the same time, these challenges may encourage greater reliance on AI tools as informal educational substitutes, offering access to information that is otherwise difficult to obtain. The implications of such use extend beyond individual learning, raising questions about digital divides, educational equity, and the risk of brain drain from already devastated health systems.

Understanding perceptions of AI among medical students requires consideration of individual and contextual factors that may shape technology acceptance. Prior literature suggests that demographic and training-related characteristics, such as gender and stage of clinical training, are associated with differing attitudes toward digital health technologies and AI adoption among medical trainees [24,25]. Additionally, prior exposure to AI tools has been consistently identified as a key determinant of perceived usefulness and acceptance within established technology acceptance frameworks [26].

Academic research experience may further influence perceptions of AI by enhancing familiarity with evidence-based practice and critical appraisal skills, potentially shaping trust and openness toward AI-supported tools in medical education and clinical contexts [26]. Accordingly, these factors were considered conceptually relevant for examining predictors of favorable AI perceptions among medical students.

This study aims to evaluate Syrian medical students’ experiences with AI tools and their perceptions regarding AI’s role in medical education and providing health care. We aim to examine whether war-related consequences—including limited computer access due to economic constraints and high emigration intentions—influence the experiences and perceptions of AI. Additionally, we aim to explore the differences across academic years to assess whether experiences and perceptions are evolving positively over time. These findings will provide insights into the readiness of Syria’s future doctors to use AI tools in the future, despite having trained in an unsupportive environment for modern technologies.

## Methods

### Study Design and Setting

This study is a cross-sectional, descriptive survey conducted in April and May 2025 among medical students and recent graduates in Syria. The primary aim was to explore their experiences with AI tools and perceptions of AI’s role in medical education and clinical practice.

### Participants and Sampling

A total of 400 participants were enrolled, including clinical-year medical students (fourth and fifth years) and preresidency students (graduates and near-graduates). Participants were recruited through convenience sampling using academic student networks and widely used social media platforms, such as closed university-affiliated groups.

Exclusion criteria were preclinical students (first to third years) and postgraduate specialty trainees (residents). Inclusion criteria included current enrollment in clinical medical training or graduation within the past 2 years, and those who voluntarily agreed to complete the questionnaire. Participation was anonymous and uncompensated.

### Note on Sampling Frame

Participants were drawn from multiple public medical faculties across various regions of Syria. The eligible population was estimated to include several thousand clinical-year students and recent graduates nationwide. Due to security and privacy considerations, specific university names are withheld.

### Data Collection Instrument

Data were collected using a structured, self-administered online questionnaire delivered via a shared Google Forms (Google LLC) link, in accordance with CHERRIES (Checklist for Reporting Results of Internet E-Surveys) guidelines. The questionnaire was originally developed in English based on published literature addressing medical students’ experiences and perceptions of AI in medical education and clinical practice. It was professionally translated into Arabic by experts in medical education and linguistics. The Arabic version was reviewed and refined by bilingual subject-matter experts to ensure linguistic accuracy, cultural appropriateness, and conceptual equivalence.

The perception items were intended to capture attitudinal views rather than to constitute a formal psychometric scale. Accordingly, internal consistency of the multi-item perception items was assessed using Cronbach α as an indicator of reliability. The final questionnaire was pilot-tested with a small group (≈30 students) to assess clarity and contextual relevance. Internal consistency of the multi-item perception scales was evaluated using Cronbach α, yielding acceptable reliability (α=.77 in pilot, α=.75 in full sample). As no modifications were made to the questionnaire following this assessment, these responses were retained and included in the final analytic dataset.

The survey consisted of 52 questions over 4 pages, organized into the following domains aligned with the Results section:

- Demographic Characteristics: age, gender, academic year, computer ownership (used as a proxy for socioeconomic status), language learning background (eg, German), and research experience.
- Prior Use of AI During Medical School: use of AI tools such as large language model–based chatbots (eg, ChatGPT) and AI-enabled visualization platforms across contexts, including study, examination preparation, clinical decision-making, and research writing.
- Intended Use of AI During Residency: anticipated or early-stage AI tool use for residency preparation and clinical reasoning among preresidency participants.
- Overall Perceptions of AI: measured via a 5-point Likert scale evaluating attitudes toward AI’s role in education, ethics, patient care, and future impact.
- Factors Influencing AI Adoption: including clinical exposure, language learning goals, emigration intentions, and access to digital technology.

### Survey Administration and Data Security

Access to the survey was restricted to researchers with password-protected accounts. The open survey link was disseminated via online academic student groups. Participation was voluntary, anonymous, and uncompensated, with no incentives offered. All questions were mandatory except for 2 optional items. A back button was available, but no summary review or adaptive questioning was used.

To protect participant confidentiality and minimize reidentification risk, no personally identifiable information (names, contact details, or institutional identifiers) or IP addresses were collected. Each Google Forms submission was treated as a unique response; duplicate or partial submissions were excluded after data cleaning.

### Data Analysis

Quantitative data were analyzed using IBM SPSS Statistics (version 27; IBM Corp). Descriptive statistics (frequencies, percentages, means, or SDs) summarized participant characteristics and AI use. Inferential analyses included:

- Chi-square tests for associations between categorical variables (eg, gender and AI usage).
- Mann-Whitney *U* tests to compare AI perception differences between subgroups (eg, gender and AI usage). Due to the nonnormal distribution of Likert-scale data, perception items were treated as ordinal variables and summarized using medians and IQRs.
- Binary logistic regression to identify predictors of positive AI perceptions and behaviors, including gender, clinical status, prior AI experience, and research background. These variables were selected a priori based on theoretical relevance and evidence from prior literature on technology acceptance in medical education. Perception outcomes were dichotomized a priori into favorable (“agree” and “strongly agree”) versus nonfavorable (all other responses). Prior AI usage experience was operationalized as a composite count variable reflecting the breadth of AI engagement. Each task was coded as present or absent and equally weighted. The total number of tasks represented cumulative prior AI exposure. Adjusted odds ratios (aORs) with 95% CIs were reported. A *P* value <.05 indicated statistical significance.

Given the exploratory nature of this study and the large number of comparisons performed, no formal adjustment for multiple comparisons was applied. Instead, exact *P* values were reported throughout to allow transparent interpretation of statistical significance.

Missing data were negligible, and all analyses were conducted using complete-case analysis without imputation.

### Ethical Considerations

Ethical approval was obtained from the Ethical Approval for Biomedical Researchers committee (1612). All participants provided informed digital consent before survey commencement, after reading an introductory consent statement outlining this study’s purpose and duration. Responses were collected anonymously and securely stored to protect privacy. Participants received no compensation for participation.

## Results

### Overview

Data collection took place between April and May 2025. By the end of the recruitment period in May 2025, a total of 400 clinical-year students and preresidency students had participated in this study. Data cleaning and verification were conducted, followed by statistical analyses completed in June 2025. The analysis focused on patterns of AI use, perceptions toward AI in medical education and patient care, and their associations with demographic characteristics. In the following analyses, the composite prior AI-use variable represents the number of different educational and research tasks for which participants reported using AI; higher scores indicate AI use across a greater number of distinct tasks.

### Demographic Characteristics

The sample was nearly gender-balanced, with 51.5% (206/400) of participants identifying as male and 48.5% (194/400) of participants as female. Most participants (336/400, 84%) were aged between 20 and 24 years. Half of the respondents were clinical-year students (200/400, 50%), and the other half were preresidency students. Notably, a large proportion (290/400, 72.5% of participants) expressed a desire to pursue postgraduate specialization abroad. In terms of technology and language exposure, 68.8% (275/400) of participants reported having access to a PC. German language study was reported by 39.8% (159/400) of participants, of whom 50.9% (81/159) of participants used AI tools to support their learning. Research experience was reported by 28.2% (113/400) of participants. Knowledge about AI was most commonly obtained through social media (294/400, 73.5% of participants), followed by reading research papers (60/400, 15% of participants) and discussions with IT-oriented peers (39/400, 9.8% participants; Table 1).

**Table 1.** Participant demographic data.

Characteristics	Participants
Gender, n (%)	
Man	206 (51.5)
Woman	194 (48.5)
Academic level, n (%)	
Clinical-years students	200 (50)
Preresidency students	200 (50)
Desire to travel abroad to complete specialization, n (%)	
Yes	290 (72.5)
No	110 (27.5)
German language study, n (%)	
Yes	159 (39.8)
No	241 (60.3)
Using AI^a^ in German language learning, n (%)	
Yes	81 (51)
No	78 (49)
PC access, n (%)	
Yes	275 (68.8)
No	125 (31.3)
Research experience, n (%)	
Yes	113 (28.2)
No	287 (71.8)
Knowing about AI, n (%)	
Social media	294 (73.5)
IT-oriented peers	39 (9.75)
Read research about it	60 (15)
See a course about it	7 (1.75)

a AI: artificial intelligence.

### Prior Use of AI During Medical School

Participants reported using AI for a variety of academic and clinical tasks. Use for studying or examination preparation was reported by 57% (228/400) of participants, while 40% (160/400) of participants used it to complete written assignments. AI was used to suggest research topics or questions by 35.5% (142/400) of participants and to assist in writing research papers by 33.8% (135/400) of participants. Additional uses included writing case reports (93/400, 23.3% participants), generating self-assessment questions (87/400, 21.8% participants), and drafting patient notes (47/400, 11.8% participants).

Use of AI-supported educational technologies during medical school was also common. Digital anatomy tools were used by 72.3% (289/400) of participants, computational pathology tools by 42.8% (171/400) of participants, and AI-generated clinical simulation cases by 22.5% (90/400) of participants (Table 2).

**Table 2.** Patterns of AI^a^ use during medical school.

Survey questions	Participants
Tasks for which AI was used, n (%)	
Assist with studying or examination preparation	228 (57)
Generate questions to test yourself	87 (21.8)
Suggest research topics or questions	142 (35.5)
Help write research papers	135 (33.8)
Help write case reports	93 (23.3)
Help write patient notes	47 (11.8)
Help complete written assignments	160 (40)
Using advanced technology or AI, n (%)	
Digital anatomy	289 (72.3)
Computational pathology	171 (42.8)
AI-generated clinical cases	90 (22.5)

a AI: artificial intelligence.

### Intended Use of AI During Residency

When asked about future use of AI during residency training, participants expressed high levels of interest. AI was considered useful for answering medical questions by 85.5% (342/400) of participants, exploring new medical topics or research by 82.8% (331/400) of participants, and supporting studying or examination preparation by 80.8% (323/400) of participants.

Further intended uses included writing research papers (300/400, 75% participants), writing case reports (261/400, 65.3% participants), writing patient notes (211/400, 52.8% participants), and assisting in clinical decision-making (208/400, 52% participants; Table 3).

**Table 3.** Intended applications of AI^a^ during residency.

Tasks for which AI will be used	Participants
Assist with studying or examination preparation, n (%)	323 (80.8)
Help answer medical questions, n (%)	342 (85.5)
Explore new medical topics or research, n (%)	331 (82.8)
Help write research papers, n (%)	300 (75)
Help write case reports, n (%)	261 (65.3)
Help write patient notes, n (%)	211 (52.8)
Assist in clinical decision-making, n (%)	208 (52)

a AI: artificial intelligence.

### Overall Perceptions of AI

#### Overview

Descriptive analysis of participant responses revealed generally favorable perceptions of AI in medical education and practice. Items were rated using a 5-point Likert scale, and positive perceptions were defined as the combined percentage of “agree” and “strongly agree” responses.

#### AI in Learning and Career Development

A total of 76% (304/400) of participants agreed that ChatGPT could improve their learning during residency, fewer participants agreed that AI tools effectively met their needs during medical school (189/400, 47.3% participants), and 48% (192/400) students preferred using ChatGPT over traditional resources such as Google or medical references.

**Figure 1.**
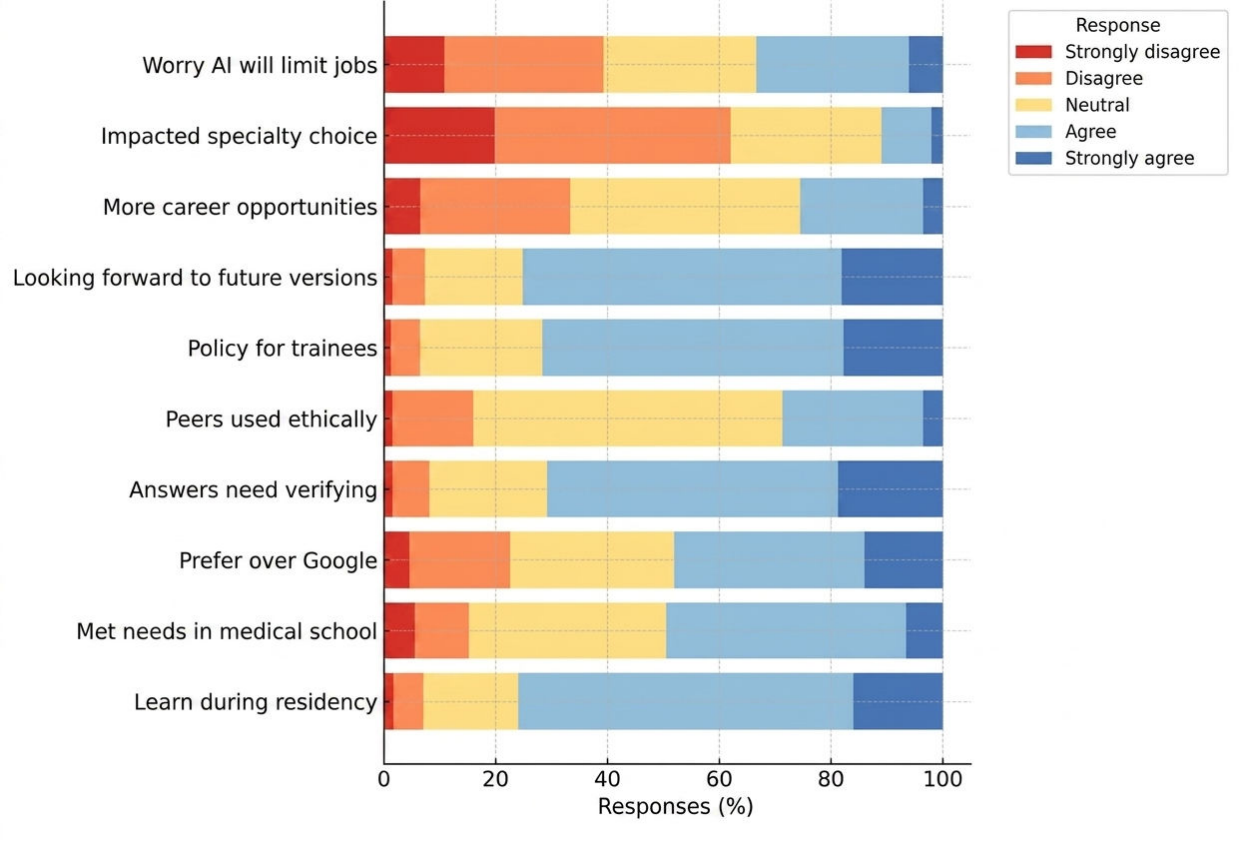
Perceptions of AI in education and career development. AI: artificial intelligence.

The need to verify AI-generated responses was endorsed by 70.8% (283/400) of participants, 75.3% (301/400) of students expressed enthusiasm for using more advanced AI systems in their future careers, and just 28.8% (115/400) students reported that their peers have always used ChatGPT ethically. Institutional regulation of AI use by trainees was supported by 71.8% (287/400) of participants. In contrast, only 25.5% (102/400) of participants believed AI would create more career opportunities, while 33.3% (133/400) of participants believed AI might limit job opportunities. AI was reported to have influenced specialty choice by 11% (44/400) of participants (Figure 1).

#### AI in Patient Care

**Figure 2.**
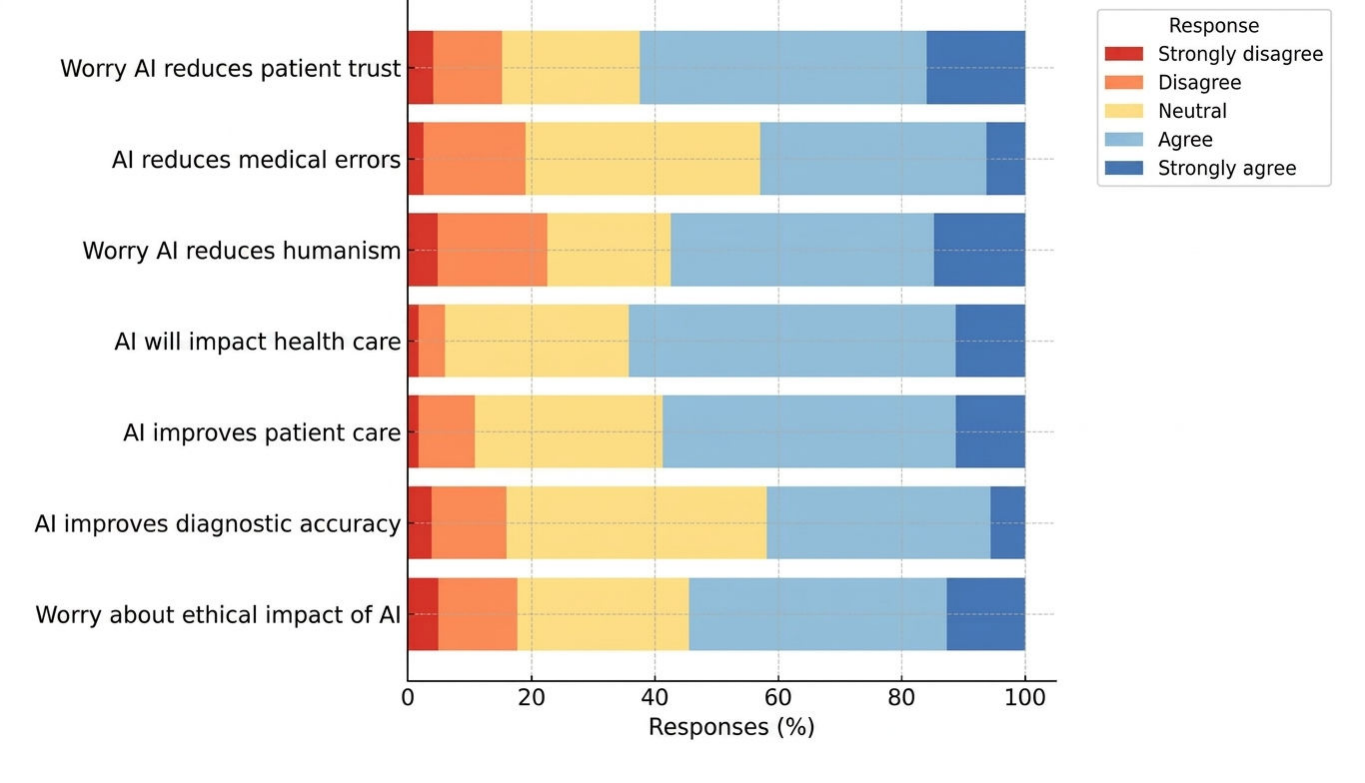
Perceptions of AI in patient care. AI: artificial intelligence.

Concerns regarding AI’s ethical impact on health care were expressed by 54.6% (218/400) of participants, and 57.6% (230/400) of participants were concerned that AI might reduce the humanistic aspect of medicine. Despite these concerns, 58.8% (235/400) of participants believed AI would enhance patient care, 42.1% (168/400) of participants believed it would improve diagnostic accuracy, and 43.1% (172/400) of participants believed it would reduce medical errors. A majority (257/400, 64.3% participants) expected AI to have a major impact on the health care system, while 62.5% (250/400) of participants expressed concern that AI might reduce patient trust in physicians (Figure 2).

### Factors Associated With AI Use

A series of chi-square tests examined associations between demographic characteristics and patterns of AI use (academic level, gender, research experience, computer availability, and German language exposure). Significant associations were identified between subgroups and multiple AI use cases (Table 4).

**Table 4.** Prior AI^a^ use by student characteristics.

AI use item and group characteristics	Values, n (%)	Chi-square (*df*)	*P* value
Assist with studying or examination preparation			
Gender		0.007 (1)	.93
Man	117 (56.8)		
Woman	111 (57.2)		
Academic level		13.219 (1)	<.001
Clinical-year student	132 (66)		
Preresidency student	96 (48)		
German language study		0.017 (1)	.90
Yes	90 (56.6)		
No	138 (57.3)		
PC access		1.855 (1)	.17
Yes	163 (59.3)		
No	65 (52)		
Research experience		0.292 (1)	.59
Yes	62 (54.9)		
No	166 (57.8)		
Generate questions to test yourself			
Gender		3.044 (1)	.08
Man	52 (25.2)		
Woman	35 (18)		
Academic level		5.303 (1)	.02
Clinical-year student	53 (26.5)		
Preresidency student	34 (17)		
German language study		0.358 (1)	.55
Yes	37 (23.3)		
No	50 (20.7)		
PC access		0.096 (1)	.76
Yes	61 (22.2)		
No	26 (20.8)		
Research experience		1.518 (1)	.22
Yes	20 (17.7)		
No	67 (23.3)		
Suggest research topics or questions			
Gender		4.258 (1)	.04
Man	83 (40.3)		
Woman	59 (30.4)		
Academic level		15.766 (1)	<.001
Clinical-year student	90 (45)		
Preresidency student	52 (26)		
German language study		0.110 (1)	.74
Yes	58 (36.5)		
No	84 (34.9)		
PC access		0.579 (1)	.45
Yes	101 (36.7)		
No	41 (32.8)		
Research experience		4.252 (1)	.04
Yes	49 (43.4)		
No	93 (32.4)		
Help write research papers			
Gender		8.128 (1)	.004
Man	83 (40.3)		
Woman	52 (26.5)		
Academic level		5.915 (1)	.02
Clinical-year student	79 (39.5)		
Preresidency student	56 (28)		
German language study		8.305 (1)	.004
Yes	67 (42.1)		
No	68 (28.2)		
PC access		7.730 (1)	.005
Yes	105 (38.2)		
No	30 (24)		
Research experience		36.896 (1)	<.001
Yes	64 (56.6)		
No	71 (24.7)		
Help write case reports			
Gender		0.249 (1)	.62
Man	50 (24.3)		
Woman	42 (22.2)		
Academic level		2.368 (1)	.12
Clinical-year student	53 (26.5)		
Preresidency student	40 (20)		
German language study		0.055 (1)	.82
Yes	36 (22.6)		
No	57 (23.7)		
PC access		1.076 (1)	.30
Yes	68 (24.7)		
No	25 (20)		
Research experience		9.506 (1)	.002
Yes	38 (33.6)		
No	55 (19.2)		
Help write patient notes			
Gender		0.311 (1)	.58
Man	26 (12.6)		
Woman	21 (10.8)		
Academic level		5.425 (1)	.02
Clinical-year student	31 (15.5)		
Preresidency student	16 (8)		
German language study		0.285 (1)	.59
Yes	17 (10.7)		
No	30 (12.4)		
PC access		1.231 (1)	.27
Yes	29 (10.5)		
No	18 (14.4)		
Research experience		1.278 (1)	.26
Yes	10 (8.8)		
No	37 (12.9)		
Help complete written assignments			
Gender		0.882 (1)	.35
Man	87 (42.2)		
Woman	73 (37.6)		
Academic level		12.042 (1)	<.001
Clinical-year student	97 (48.5)		
Preresidency student	63 (31.5)		
German language study		5.653 (1)	.02
Yes	75 (47.2)		
No	85 (35.3)		
PC access		0.436 (1)	.51
Yes	113 (41.1)		
No	47 (37.6)		
Research experience		0.403 (1)	.53
Yes	48 (42.5)		
No	112 (39)		
Digital anatomy			
Gender		5.158 (1)	.02
Man	159 (77)		
Woman	130 (67)		
Academic level		0.012 (1)	.91
Clinical-year student	145 (72.5)		
Preresidency student	144 (72)		
German language study		4.333 (1)	.04
Yes	124 (78)		
No	165 (68.5)		
PC access		10.286 (1)	<.001
Yes	212 (77.1)		
No	77 (61.6)		
Research experience		10.976 (1)	<.001
Yes	95 (84.1)		
No	194 (67.6)		
Computational pathology			
Gender		1.049 (1)	.31
Man	83 (40.3)		
Woman	88 (45.4)		
Academic level		0.255 (1)	.613
Clinical-year student	88 (44)		
Preresidency student	83 (41.5)		
German language study		0.692 (1)	.41
Yes	72 (45.3)		
No	99 (41.1)		
PC access		1.406 (1)	.24
Yes	123 (44.7)		
No	48 (38.4)		
Research experience		1.110 (1)	.29
Yes	53 (46.9)		
No	118 (41.1)		
AI-generated cases for simulation			
Gender		1.086 (1)	.30
Man	42 (20.4)		
Woman	48 (24.7)		
Academic level		4.645 (1)	.03
Clinical-year student	54 (27)		
Preresidency student	36 (18)		
German language study		0.090 (1)	.76
Yes	37 (23.3)		
No	53 (22)		
PC access		1.135 (1)	.29
Yes	66 (24)		
No	24 (19.2)		
Research experience		0.144 (1)	.71
Yes	24 (21.2)		
No	66 (23)		

a AI: artificial intelligence.

Clinical-year students reported higher use of AI for studying or examination preparation compared with preresidency students (66% vs 48%; *χ*²_1_=13.21; *P*<.001). Similar differences were observed for generating self-assessment questions (26.5% vs 17%, *χ*²_1_=5.30; *P*=.02), suggesting research topics (45% vs 26%, *χ*²_1_=15.76; *P*<.001), writing research papers (39.5% vs 28%, *χ*²_1_=5.91; *P*=.02), completing written assignments (48.5% vs 31.5%, *χ*²_1_=12.04; *P*<.001), creating simulated clinical cases (27% vs 18%, *χ*²_1_=4.64; *P*=.03), and writing patient notes (15.5% vs 8%, *χ*²_1_=5.42; *P*=.02).

Gender differences were observed across several AI-use domains. Male participants reported greater use of AI for writing research papers (40.3% vs 26.8%; *χ*²_1_=8.12; *P*=.004), suggesting research topics (40.3% vs 30.4%; *χ*²_1_=4.25; *P*=.04), and using digital anatomy tools (77.2% vs 67%; *χ*²_1_=5.15; *P*=.02).

Participants with research experience were significantly more likely to use AI for suggesting research topics (43.4% vs 32.4%; *χ*²_1_=4.25; *P*=.04), writing research papers (56.6% vs 24.7%; *χ*²_1_=36.89; *P*<.001), writing case reports (33.6% vs 19.2%; *χ*²_1_=9.50; *P*=.002), and using digital anatomy platforms (84.1% vs 67.6%; *χ*²_1_=10.97; *P*<.001).

Access to a PC was associated with greater use of AI for writing research papers (38.2% vs 24%; *χ*²_1_=7.73; *P*=.005) and for learning via digital anatomy platforms (77.1% vs 61.6%; *χ*²_1_=10.28; *P*<.001).

Students studying German reported slightly higher use of AI for language learning and for completing written assignments (47.2% vs 35.3%; *χ*²_1_=5.65; *P*=.02) and to help write research papers (42.1% vs 28.2%; *χ*²_1_=8.30; *P*=.004). This trend reflects a culturally motivated behavior, particularly among Syrian students, who often aspire to pursue postgraduate training in Germany due to the ongoing crisis. These students appeared to use AI to simulate German language acquisition, highlighting AI’s potential role in educational mobility and international preparation.

### Factors Associated With Perceptions of AI for Career and Education

Perceptions of AI for career and educational domains were compared across student subgroups (academic level, gender, research experience, access to a PC, and German language exposure) using Mann-Whitney *U* tests.

Preresidency students reported higher agreement that ChatGPT improves learning during residency compared with clinical-year students (median 4, IQR 4‐4, vs 4, IQR 3‐4; U=17,736; *P*=.03). They also showed stronger agreement that medical schools and residency programs should establish formal policies regulating AI use by trainees (median 4, IQR 4‐4, vs 4, IQR 3‐4; U=17,497; *P*=.02). In contrast, clinical-year students rated AI as more effective in meeting their needs during medical school than preresidency students (median 4, IQR 3‐4, vs 3, IQR 3‐4; U=16,501; *P*<.001).

Gender-based differences were observed across several items. Male students reported higher ratings for AI supporting learning during residency (median 4, IQR 4‐4, vs 4, IQR 3‐4; U=17,254.5; *P*=.007), preference for ChatGPT over other search engines (median 4, IQR 3‐4, vs 3, IQR 2‐4; U=16,667; *P*=.003), and enthusiasm toward future versions of AI (median 4, IQR 4‐4, vs 4, IQR 3‐4; U=17,264; *P*=.009).

Students without PCs reported greater influence of AI on residency specialty choice compared with those with computers (median 3, IQR 2‐3, vs 3, IQR 2‐3; U=15,059; *P*=.04). Conversely, students with PCs were less likely to agree that their peers consistently used AI ethically (median 3, IQR 3‐3, vs 3, IQR 3‐4; U=14,595; *P*=.007).

Students who had learned German rated AI as more helpful for learning during residency than those who had not (median 4, IQR 4‐4, vs 4, IQR 3‐4; U=16,350; *P*=.005), which may reflect AI’s perceived value in language acquisition for migration-preparing students. Additionally, they showed enthusiasm toward future versions of AI (median 4, IQR 4‐5, vs 4, IQR 3‐4; U=16,136; *P*=.003).

Research experience was also associated with perception differences. Students with research experience were less confident in the accuracy of AI’s outputs (median 4, IQR 4‐5, vs 4, IQR 3‐4; U=13,042; *P*<.001), more supportive of institutional AI policies (median 4, IQR 4‐4.5, vs 4, IQR 3‐4; U=13,535; *P*=.005), and more likely to agree that AI improves learning during residency (median 4, IQR 4‐4, vs 4, IQR 3‐4; U=14,272; *P*=.03). In contrast, students without research experience were more likely to perceive their peers as using AI ethically (median 3, IQR 3‐4, vs 3, IQR 2‐4; U=13,902; *P*=.01) and to report greater influence of AI on residency specialty choice (median 2, IQR 2‐3, vs 2, IQR 1‐3; U=14,175; *P*=.04). Detailed Mann-Whitney *U* test results for perceptions of AI in education and career are provided in Multimedia Appendix 1.

### Factors Associated With Perceptions of AI for Patient Care

A series of Mann-Whitney *U* tests were conducted to compare perceptions of AI for patient care and professional practice among medical students’ subgroups (academic level, gender, research experience, access to a PC, and German language exposure).

Gender again played an important role where male students reported significantly higher agreement that AI will improve diagnostic accuracy compared with female students (median 4, IQR 3‐4, vs 3, IQR 3‐4; U=15,489; *P*<.001), improve patient care (median 4, IQR 3‐4, vs 4, IQR 3‐4; U=15,281; *P*<.001), AI will have a major impact on health care (median 4, IQR 3‐4, vs 4, IQR 3‐4; U=16,171; *P*<.001), reduce medical errors (median 3, IQR 3‐4, vs 3, IQR 3‐4; U=16,931; *P*=.005), whereas female students worry more about the ethical impact of AI on health care (median 4, IQR 3‐4, vs 3, IQR 3‐4; U=17,410; *P*=.02).

Academic level was also associated with differences in perception. Preresidency students believed more that AI will have a major impact on health care during residency (median 4, IQR 3‐4, vs 4, IQR 3‐4; U=17,760; *P*=.03).

German language learners expressed higher agreement with the fact that AI will improve patient care (median 4, IQR 3‐4, vs 4, IQR 3‐4; U=16,308; *P*=.007), have a major impact on health care (median 4, IQR 3‐4, vs 4, IQR 3‐4; U=16,680; *P*=.02) and will enable them to make more accurate diagnoses (median 4, IQR 3‐4, vs 3, IQR 3‐4; U=15,716; *P*<.001).

Research experience was associated with increased concern that AI will reduce patient trust in physicians (median 4, IQR 3‐4, vs 4, IQR 3‐4; U=14,072; *P*=.03). Detailed Mann-Whitney *U* test results for perceptions of AI in patient care are available in Multimedia Appendix 2.

### Predictors of Positive AI Perceptions

Multivariable binary logistic regression analyses were conducted to identify predictors of positive perceptions of AI across educational and clinical domains. Prior AI usage experience significantly increased the odds of a favorable perception in multiple domains: each additional AI‐use task raised the likelihood that students would agree that AI improves learning during residency (aOR=1.43, 95% CI 1.15‐1.79, *P*=.002), that AI was effective in meeting medical‐school needs (aOR=1.60, 95% CI 1.34‐1.92, *P*<.001), and that it would help reduce medical errors (aOR=1.14, 95% CI 1.01‐1.28, *P*=.04).

Clinical‐year status was consistently associated with more reserved views: clinical-year students were associated with lower odds of perceiving AI as beneficial for residency learning (aOR=0.26, 95% CI 0.11‐0.64, *P*=.003), less likely to anticipate using future versions (aOR=0.18, 95% CI 0.07‐0.49, *P*<.001), and less likely to expect AI to have a major impact on health care (aOR=0.24, 95% CI 0.09‐0.65, *P*=.005).

Gender differences appeared: female students had 3 times the odds of insisting on verifying AI’s answers (aOR=3.12, 95% CI 1.36‐7.15, *P*=.007) and female students were twice as likely to worry about AI’s ethical impact on health care (aOR=2.06, 95% CI 1.19‐3.54, *P*=.01).

Research experience did not significantly predict any positive perception after adjustment.

## Discussion

### Principal Findings

This multicenter cross-sectional survey of 400 medical students and recent graduates in the Syrian Arab Republic addresses a notable gap in the literature on AI in medical education in a low-resource, conflict-affected setting. We found that students increasingly rely on AI tools for educational and clinical purposes, with the highest reported uses being examination preparation (228/400, 57%), written assignments (160/400, 40%), and digital anatomy platforms (289/400, 72.3%). Usage patterns differed significantly by academic level (higher use among clinical-year students), gender, computer access, and research experience. Although participants were generally optimistic about AI tools’ potential to enhance learning and residency training, many expressed ethical concerns and worries about reduced humanistic aspects of care; only 25.5% (102/400) of students expected direct career benefits. These findings both demonstrate the growing role of AI tools among Syrian medical trainees and underscore the need for institutional policies, targeted training, and efforts to address digital access gaps to ensure ethical and equitable integration. Thus, the summary of key findings is the high uptake of AI for study and anatomy tools; generally positive but ethically cautious perceptions; and notable subgroup differences by academic level, gender, access, and research experience.

### Methodological Reminder

The cross-sectional design of this study limits causal inference; therefore, interpretations about drivers of use are based on observed associations rather than proven causal relationships.

### Contextual Linkage

#### Overview

The national context—war-related damage, economic collapse, recurrent power cuts, and high emigration intentions—plausibly shapes patterns of access and attitudes reported here and helps explain some observed associations (eg, device ownership, language study, and intentions to train abroad). These contextual factors may influence who can access AI tools, who invests in language skills for migration, and which trainees consider leaving for postgraduate opportunities; we discuss these implications below.

#### Prior Use of AI During Medical School

A total of 57% (228/400) of participants reported using AI tools for examination preparation, 40% (160/400) of participants for written assignments, and 72.3% (289/400) of participants used digital anatomy tools. Further, 94% (376/400) of participants reported prior exposure to AI tools. These core results indicate high uptake of AI tools for study and academic tasks among our sample. When comparing with prior studies, reported rates vary by context and timing; we therefore present comparisons cautiously and note contextual differences (eg, study populations, timing, and local resources). For example, our experience figure is higher than a previous Syrian report (70%) [27], and our examination preparation rate (57%) exceeds a US study reporting that 48.9% of medical students use AI tools for study support [28]. Use for written assignments (40%) is higher than a UAE residency applicant sample (20.4%) [29], and use for research writing (33.8%) compares with the 24.8% in a Ugandan sample and 36.6% in a Malaysian study [30,31]. These contextual differences (population, timing, infrastructure, and educational policy) may explain variation between studies and caution against direct equivalence. Digital anatomy use (72.3%) is similar to a Canadian report of 68.1% [32].

#### Intended Use of AI During Residency

AI tools were reported by 85.5% (342/400) of participants as tools they would use to answer medical questions, and 82.8% (331/400) of participants indicated they would use AI tools to explore emerging medical topics or conduct research. These figures reflect intended or future use among students and recent graduates rather than measured practice among current residents. In contrast, another study among medical students revealed that 73% used AI to inquire about medical knowledge [33]. Similarly, in our study, 52% (208/400) of participants reported they would use AI tools to assist in clinical decision-making. Moreover, previous research has consistently demonstrated that some AI systems achieved high performance in certain diagnostic tasks, with reported accuracies in specific settings [34]; such performance metrics should be interpreted in context and do not imply direct transferability to routine clinical practice.

### Factors Associated With AI Use

Analysis of the data reveals that the usage rate of AI tools was notably higher among males than among females across almost all fields, a pattern that aligns with findings from studies conducted in Sudan and the United Arab Emirates [35,36]. Moreover, this higher usage phenomenon may reflect differences in attitudes, access, or self-reported behaviors rather than causal effects; we therefore describe these as associations rather than causes [37]. In addition, clinical students consistently demonstrated a greater likelihood of using AI tools across many tasks compared to both graduate and prospective students, reinforcing the notion that different academic cohorts adopt AI at varying intensities. Higher AI use among clinical students may reflect greater curricular demands or exposure; qualitative work is needed to confirm underlying reasons. However, our findings stand in contrast to a study conducted in China, which reported that graduate medical students exhibited greater awareness of medical AI usage than undergraduate students [38]. To examine socioeconomic influences, we used ownership of a PC as a proxy; access to a PC was positively associated with AI usage—an observation consistent with findings from Saudi Arabia [39].

### Contextual Interpretation of Specific Variables

First, computer ownership likely reflects unequal access to digital infrastructure within the country; in the context of economic hardship and power instability, some students may lack reliable devices or connectivity, widen digital divides, and limit opportunities to use AI tools. Second, the association between German language study and higher AI use may reflect preparatory behavior among students aiming for postgraduate training abroad, who invest in language and digital tools to improve competitiveness. Third, intentions to specialize abroad raise concerns about potential brain drain: if those with greater access and digital literacy are also more likely to emigrate, local inequities in human resources for health could be exacerbated.

Although learning the German language was not statistically associated with most AI applications, students studying German reported slightly higher use of AI tools for language learning, particularly for completing written assignments (*P*=.02), and they used AI tools more frequently to assist with writing research papers than students not studying German. Previous studies have demonstrated that AI significantly impacts students’ language learning [40]. This trend may reflect culturally motivated behavior among students preparing for postgraduate training abroad and highlights AI tools’ potential role in facilitating language acquisition and international mobility.

### Perceptions of AI Tools for Career, Education, and Patient Care

#### Overview

A total of 76% (304/400) of respondents indicated that AI tools can enhance learning outcomes, whereas an Indian study reported that 91.3% believed it could improve educational efficiency [41]. Educational policies, internet accessibility, and medical training approaches may all shape these opinions. Syrian students may face resource constraints that limit the practical benefits they can derive from AI tools. A total of 57.6% (230/400) of participants expressed concern that AI tools might diminish the human aspect of medicine. Despite technological advancements, the human touch remains a vital component of health care. AI tools should be regarded as tools to support compassionate care, rather than replace it [42]. Some students expressed concern about the potential impact of AI tools on the medical labor market. Further, 11% (44/400) of participants indicated that AI influenced their choice of specialty—a figure notably lower than the 25% reported in a study conducted in Ontario [43]. This may reflect the early stage of AI integration in Syrian clinical education or competing priorities, such as employment and migration. A total of 62.5% (250/400) of participants expressed concern that AI tools might reduce patients’ trust in physicians, in contrast to a study conducted in Croatia and Slovakia, where 51.3% of students believed that patients would trust physicians less [44]. This difference may be attributed to the perception that the personal relationship between doctor and patient in Syria is deeply human and built on personal trust; therefore, the introduction of a technological element may be viewed as a direct threat to this conventional relationship. Despite some evident concerns—particularly regarding ethics and the human dimension of care—most students generally expressed optimism about integrating AI tools into both educational and clinical settings. Some skepticism existed regarding the ability of AI to enhance diagnostic accuracy, with only 42.1% (168/400) of participants agreeing to this notion. In comparison, a study conducted in London, United Kingdom, found that only 46% agreed that AI would improve clinical judgment [45]. This suggests that medical students do not view AI tools as a magical tool for enhancing diagnostics, but rather as a promising technology that should complement—rather than replace—human clinical reasoning. Approximately 33.3% (133/400) of respondents agreed that AI would limit job opportunities. In contrast, a study in Canada revealed that 98% of participants believed AI had the potential to replace some, most, or all medical activities in the field of radiation oncology [46]. This disparity may indicate that Syrian students view AI as an emerging technology designed to complement medical practice rather than to threaten it, or they might not have yet experienced practical demonstrations that reveal the autonomous capabilities of this technology.

#### Factors Associated With Perceptions of AI for Career and Education

Graduate and prospective graduate students expressed significantly more positive perceptions regarding AI tools’ usefulness in learning during residency (*P*=.03). This finding suggests that AI is regarded as a valuable educational aid during clinical rotations. We attribute these perceptions to their increased exposure to clinical practice and heightened awareness of the educational challenges that AI tools can help address (eg, high caseloads, difficulties in accessing references, and time constraints). This finding is consistent with a study involving 1243 undergraduate and graduate students from 13 universities and 33 hospitals, which found that graduate medical students demonstrated a higher level of awareness regarding the use of medical AI than their undergraduate counterparts [38]. Moreover, these students were more inclined than clinical-year students to believe that medical schools and residency programs should develop policies regarding the use of AI tools by trainees (*P*=.02). This trend reflects their advanced awareness of the professional responsibilities and organizational challenges associated with these tools.

Male students rated AI significantly higher in supporting residency learning (*P*=.007), preference for ChatGPT over other search engines (*P*=.003), and enthusiasm toward future versions of AI (*P*=.009). This may indicate that male students hold a stronger perception of the role of AI in developing clinical skills and preparing for postgraduate education.

The perceived potential of AI appeared to influence residency specialty choices more among students without PCs (*P*=.04). This may be attributed to their increased reliance on AI tools accessible via smartphones or public devices, which can serve as critical supports in making professional decisions when personal technological resources are limited. In contrast, students with PCs were more likely to believe that their peers had used ChatGPT less ethically than students without such resources (*P*=.007). This perception may stem from their deeper understanding of how these tools are used or from greater exposure to ethical discussions regarding the appropriate use of AI in research and academic tasks.

Those with experience were less confident in the accuracy of AI tools (*P*<.001), possibly due to their direct encounter with its shortcomings or biases, which led them to adopt a more cautious stance. They were also more supportive of medical schools that implement policies for the use of AI (*P*=.005), a perspective likely attributable to their awareness of the risks associated with unregulated use. Moreover, they held a more positive view regarding the utility of ChatGPT in supporting learning during residency (*P*=.03), reflecting their detailed understanding of its potential to enhance clinical education when applied within an appropriate framework. Additionally, among those without experience, residency choices were more influenced by the perceived capabilities of AI tools (*P*=.04), which may reflect a theoretical enthusiasm rather than one grounded in direct experience.

#### Factors Associated With Perceptions of AI for Patient Care

Gender also played a significant role. Male students demonstrated markedly higher agreement that AI tools would improve diagnostic accuracy (*P*<.001), enhance patient care (*P*<.001), exert a substantial impact on health care (*P*<.001), and reduce medical errors (*P*=.005). This finding is consistent with a study conducted in the United Arab Emirates, which reported that male students were more inclined than female students to believe that AI improves diagnostic accuracy (51.7% vs 39.7%), reduces medical errors (58.2% vs 40.8%), and enhances patient care (65.9% vs 54.6%) [29]. In contrast, female students expressed greater concern about the ethical implications of AI in health care (*P*=.02), reflecting their heightened sensitivity to the humanistic aspects of medical practice. This concern may be associated with fears of diminished human interaction or reduced trust in technology within clinical contexts.

German-language learners expressed significantly higher agreement with the statements that AI tools would improve patient care (*P*=.007), have a substantial impact on health care (*P*=.02), and enable more accurate diagnoses (*P*=.001). This may be related to the content of German-language medical programs or the medical culture associated with the German-speaking context, which may place greater emphasis on modern technologies and their clinical implications. Research experience was associated with greater concern that AI tools could diminish patients’ trust in physicians (*P*=.03), possibly reflecting heightened awareness of ethical and trust issues among those engaged in research.

### Limitations

While this study offers insights into AI tools’ perceptions and use among Syrian medical trainees, limitations include convenience sampling, reliance on self-reported data subject to recall and social desirability bias, use of proxy variables (eg, computer ownership for socioeconomic status), and lack of prestudy sample size calculation; these factors limit generalizability and preclude causal inference. We explicitly note that PC ownership was used as a proxy for income; however, this is an imperfect and 1D indicator that does not capture the multidimensional nature of socioeconomic status. The cross-sectional design and self-reported measures limit causal inference; hypotheses about drivers of AI tool use require longitudinal or qualitative validation. Further, this study involved a large number of statistical comparisons across multiple domains. Although no formal adjustment for multiple comparisons was applied, exact *P* values were reported throughout to support transparent interpretation of the findings, and results with marginal statistical significance should be interpreted with caution. Additionally, items referring to “during residency” capture intended future use for most respondents and do not measure observed practice among current residents.

### Further Studies

Future research should consider longitudinal or mixed method designs to better understand evolving perceptions of AI tools throughout medical training and early clinical practice. Qualitative methodologies, including interviews and focus groups, could deepen insights into the reasoning behind student attitudes—especially regarding trust, ethics, and specialty choices. Expanding the sample to include students from private institutions, rural areas, or international campuses would improve the representativeness of findings. Policymakers and educators should consider equity-focused interventions that address device and connectivity gaps and strategies to retain trained graduates to mitigate potential brain drain.

### Conclusions

This study reveals a predominantly optimistic outlook among Syrian medical students and residents regarding the role of AI in education and clinical care. High levels of prior usage suggest both accessibility and growing technological literacy, though notable concerns—particularly ethical and relational—underscore the need for guided integration. Differences across gender, training stage, and socioeconomic proxies reflect the nuanced landscape of AI perception in medical education. While enthusiasm is widespread, cautious appraisal of AI’s limitations and its potential to disrupt traditional human-centered care remains essential. As such, integrating AI into medical education must be performed strategically, emphasizing critical appraisal, ethical awareness, and reinforcement of compassionate clinical reasoning. These findings contribute meaningfully to the regional and global discourse on responsible AI adoption in health professions education.

## Supplementary material

10.2196/84942 Multimedia Appendix 1 Detailed Mann-Whitney U test results for perceptions of AI in education and career. AI: artificial intelligence.

10.2196/84942 Multimedia Appendix 2 Detailed Mann-Whitney U test results for perceptions of AI in patient care. AI: artificial intelligence.

10.2196/84942 Checklist 1 CHERRIES Checklist.

## Abbreviations

AI: artificial intelligence
aOR: adjusted odds ratio
CHERRIES: Checklist for Reporting Results of Internet E-Surveys
USMLE: United States Medical Licensing Examination
