# Supplementary material for: Experiences and Perceptions of Clinical and Graduate Medical Students Regarding AI in Syria: Cross-Sectional Study
Source: JMIR Med Educ. 2026 May 19;12:e84942. doi: 10.2196/84942 (PMC13186531; doi:10.2196/84942)
Supplement: Multimedia Appendix 1 — Detailed Mann-Whitney U test results for perceptions of AI in education and career. AI: artificial intelligence. [file mededu-v12-e84942-s001.docx]

| Perception item | Group characteristic | | Median (IQR) | Mann-Whitney U | P value |
| --- | --- | --- | --- | --- | --- |
| AI will improve my ability to learn during my residency | Gender | |  | | |
|  |  | Male | 4 (4-4) | 17254.5 | **.007** |
|  |  | Female | 4(3-4) |  |  |
|  | Academic level | |  | | |
|  |  | Clinical-year student | 4(3-4) | 17736.0 | **.026** |
|  |  | Pre-residency student | 4(4-4) |  |  |
|  | German language study | |  | | |
|  |  | Yes | 4(4-4) | 16350.5 | **.005** |
|  |  | No | 4(3-4) |  |  |
|  | Personal computer access | |  | | |
|  |  | Yes | 4(4-4) | 16963.5 | .812 |
|  |  | No | 4(4-4) |  |  |
|  | Prior research experience | |  |  |  |
|  |  | Yes | 4(4-4) | 14272.5 | **.034** |
|  |  | No | 4(3-4) |  |  |
| AI was effective in meeting my needs during medical school | Gender | |  | | |
|  |  | Male | 3(3 – 4) | 18583.0 | .195 |
|  |  | Female | 3(3 – 4) |  |  |
|  | Academic level | |  | | |
|  |  | Clinical-year student | 4(3 – 4) | 16501.0 | **.001** |
|  |  | Pre-residency student | 3(3 – 4) |  |  |
|  | German language study | |  | | |
|  |  | Yes | 3(3 – 4) | 18899.5 | .806 |
|  |  | No | 3(3 – 4) |  |  |
|  | Personal computer access | |  | | |
|  |  | Yes | 4(3 – 4) | 15918.5 | .205 |
|  |  | No | 3(3 – 4) |  |  |
|  | Prior research experience | |  |  |  |
|  |  | Yes | 3(3-4) | 15898.0 | .744 |
|  |  | No | 3(3-4) |  |  |
| I prefer using ChatGPT rather than Google or another search engine or medical reference to explain a medical topic | Gender | |  | | |
|  |  | Male | 4 (3-4) | 16667.0 | **.003** |
|  |  | Female | 3(2-4) |  |  |
|  | Academic level | |  | | |
|  |  | Clinical-year student | 3(3-4) | 19971.0 | .997 |
|  |  | Pre-residency student | 3(3-4) |  |  |
|  | German language study | |  | | |
|  |  | Yes | 3(3-4) | 18983.5 | .872 |
|  |  | No | 3(3-4) |  |  |
|  | Personal computer access | |  | | |
|  |  | Yes | 3(3-4) | 15727.5 | .157 |
|  |  | No | 4(3-4) |  |  |
|  | Prior research experience | |  |  |  |
|  |  | Yes | 3(2-4) | 15564.5 | .516 |
|  |  | No | 3(3-4) |  |  |
| The answers provided by AI need to be verified | Gender | |  | | |
|  |  | Male | 4 (3-4) | 19144.0 | .43 |
|  |  | Female | 4(3-4) |  |  |
|  | Academic level | |  | | |
|  |  | Clinical-year student | 4(3-4) | 19062.0 | .377 |
|  |  | Pre-residency student | 4(3-4) |  |  |
|  | German language study | |  | | |
|  |  | Yes | 4(3-4) | 18729.0 | .679 |
|  |  | No | 4(3-4) |  |  |
|  | Personal computer access | |  | | |
|  |  | Yes | 4(3-4) | 16871.5 | .748 |
|  |  | No | 4(3-4) |  |  |
|  | Prior research experience | |  |  |  |
|  |  | Yes | 4(4-5) | 13042.5 | **<.001** |
|  |  | No | 4(3-4) |  |  |
| My peers have always used AI ethically | Gender | |  | | |
|  |  | Male | 3(3-3) | 18286.0 | .103 |
|  |  | Female | 3(3-4) |  |  |
|  | Academic level | |  | | |
|  |  | Clinical-year student | 3(3-4) | 18913.5 | **.**297 |
|  |  | Pre-residency student | 3(3-4) |  |  |
|  | German language study | |  | | |
|  |  | Yes | 3(3-4) | 17851.5 | .200 |
|  |  | No | 3(3-4) |  |  |
|  | Personal computer access | |  | | |
|  |  | Yes | 3(3-3) | 14595.0 | **.007** |
|  |  | No | 3(3-4) |  |  |
|  | Prior research experience | |  |  |  |
|  |  | Yes | 3(2-4) | 13902.0 | **.014** |
|  |  | No | 3(3-4) |  |  |
| Medical schools and residency programs should develop policies about the use of AI by trainees | Gender | |  | | |
|  |  | Male | 4 (3-4) | 19913.0 | **.**948 |
|  |  | Female | 4(3-4) |  |  |
|  | Academic level | |  | | |
|  |  | Clinical-year student | 4(3-4) | 17497.0 | **.017** |
|  |  | Pre-residency student | 4(4-4) |  |  |
|  | German language study | |  | | |
|  |  | Yes | 4(3-4) | 19074.0 | .934 |
|  |  | No | 4(3-4) |  |  |
|  | Personal computer access | |  | | |
|  |  | Yes | 4(3-4) | 15598.0 | .103 |
|  |  | No | 4(3-4) |  |  |
|  | Prior research experience | |  |  |  |
|  |  | Yes | 4(4-4.5) | 13535.5 | **.005** |
|  |  | No | 4(3-4) |  |  |
| I am looking forward to using newer versions of AI in my career | Gender | |  | | |
|  |  | Male | 4 (4-4) | 17264.0 | **.009** |
|  |  | Female | 4(3-4) |  |  |
|  | Academic level | |  | | |
|  |  | Clinical-year student | 4(3-4) | 18412.0 | .125 |
|  |  | Pre-residency student | 4(4-4) |  |  |
|  | German language study | |  | | |
|  |  | Yes | 4(4-5) | 16136.0 | **.003** |
|  |  | No | 4(3-4) |  |  |
|  | Personal computer access | |  | | |
|  |  | Yes | 4(3-4) | 16968.5 | .819 |
|  |  | No | 4(4-4) |  |  |
|  | Prior research experience | |  |  |  |
|  |  | Yes | 4(3-5) | 15179.5 | 0.266 |
|  |  | No | 4(4-4) |  |  |
| AI will create more career opportunities for me as a physician | Gender | |  | | |
|  |  | Male | 3(2-3) | 19091.0 | .416 |
|  |  | Female | 3(2-4) |  |  |
|  | Academic level | |  | | |
|  |  | Clinical-year student | 3(2-3) | 19337.0 | .546 |
|  |  | Pre-residency student | 3(2-4) |  |  |
|  | German language study | |  | | |
|  |  | Yes | 3(2-4) | 18259.5 | .402 |
|  |  | No | 3(2-3) |  |  |
|  | Personal computer access | |  | | |
|  |  | Yes | 3(2-4) | 15910.5 | .209 |
|  |  | No | 3(2.5-3) |  |  |
|  | Prior research experience | |  |  |  |
|  |  | Yes | 3(2-4) | 15242.5 | .324 |
|  |  | No | 3(2-3) |  |  |
| AI’s potential has impacted my residency specialty choice | Gender | |  | | |
|  |  | Male | 2(2-3) | 19933.0 | .964 |
|  |  | Female | 2(2-3) |  |  |
|  | Academic level | |  | | |
|  |  | Clinical-year student | 2(2-3) | 18218.0 | .104 |
|  |  | Pre-residency student | 2(2-3) |  |  |
|  | German language study | |  | | |
|  |  | Yes | 2(2-3) | 18585.0 | .592 |
|  |  | No | 2(2-3) |  |  |
|  | Personal computer access | |  | | |
|  |  | Yes | 2(2-3) | 15059.0 | **.036** |
|  |  | No | 2(2-3) |  |  |
|  | Prior research experience | |  |  |  |
|  |  | Yes | 2(1-3) | 14175.5 | **.038** |
|  |  | No | 2(2-3) |  |  |
| I worry that AI will limit my job options in the future | Gender | |  | | |
|  |  | Male | 3(2-4) | 17882.0 | .060 |
|  |  | Female | 3(2-4) |  |  |
|  | Academic level | |  | | |
|  |  | Clinical-year student | 3(2-4) | 19672.0 | .769 |
|  |  | Pre-residency student | 3(2-4) |  |  |
|  | German language study | |  | | |
|  |  | Yes | 3(2-4) | 18777.0 | .727 |
|  |  | No | 3(2-4) |  |  |
|  | Personal computer access | |  | | |
|  |  | Yes | 3(2-4) | 16996.0 | .853 |
|  |  | No | 3(2-4) |  |  |
|  | Prior research experience | |  |  |  |
|  |  | Yes | 3(2-4) | 16196.5 | .985 |
|  |  | No | 3(2-4) |  |  |
