# Supplementary material for: Experiences and Perceptions of Clinical and Graduate Medical Students Regarding AI in Syria: Cross-Sectional Study
Source: JMIR Med Educ. 2026 May 19;12:e84942. doi: 10.2196/84942 (PMC13186531; doi:10.2196/84942)
Supplement: Multimedia Appendix 2 — Detailed Mann-Whitney U test results for perceptions of AI in patient care. AI: artificial intelligence. [file mededu-v12-e84942-s002.docx]

| Perception item | Group characteristic | | Median (IQR) | Mann-Whitney U | P value |
| --- | --- | --- | --- | --- | --- |
| I worry about the ethical impact of AI on healthcare | Gender | |  | | |
|  |  | Male | 3(3-4) | 17410.0 | **.019** |
|  |  | Female | 4(3-4) |  |  |
|  | Academic level | |  | | |
|  |  | Clinical-year student | 3.5(3-4) | 17864.0 | **.052** |
|  |  | Pre-residency student | 4(3-4) |  |  |
|  | German language study | |  | | |
|  |  | Yes | 4(3-4) | 18263.5 | .404 |
|  |  | No | 4(3-4) |  |  |
|  | Personal computer access | |  | | |
|  |  | Yes | 4(3-4) | 16555.0 | .534 |
|  |  | No | 4(3-4) |  |  |
|  | Prior research experience | |  |  |  |
|  |  | Yes | 4(3-4) | 15394.0 | .406 |
|  |  | No | 4(3-4) |  |  |
| AI will enable me to make more accurate diagnoses as a physician | Gender | |  | | |
|  |  | Male | 4(3-4) | 15489.5 | **<.001** |
|  |  | Female | 3(3-4) |  |  |
|  | Academic level | |  | | |
|  |  | Clinical-year student | 3(3-4) | 18388.0 | .136 |
|  |  | Pre-residency student | 3(3-4) |  |  |
|  | German language study | |  | | |
|  |  | Yes | 4(3-4) | 15716.5 | **.001** |
|  |  | No | 3(3-4) |  |  |
|  | Personal computer access | |  | | |
|  |  | Yes | 3(3-4) | 17094.5 | .926 |
|  |  | No | 3(3-4) |  |  |
|  | Prior research experience | |  |  |  |
|  |  | Yes | 3(3-4) | 16072.5 | .883 |
|  |  | No | 3(3-4) |  |  |
| AI will improve patient care during my career | Gender | |  | | |
|  |  | Male | 4(3-4) | 15281.5 | **<.001** |
|  |  | Female | 4(3-4) |  |  |
|  | Academic level | |  | | |
|  |  | Clinical-year student | 4(3-4) | 18544.0 | .175 |
|  |  | Pre-residency student | 4(3-4) |  |  |
|  | German language study | |  | | |
|  |  | Yes | 4(3-4) | 16308.5 | **.007** |
|  |  | No | 4(3-4) |  |  |
|  | Personal computer access | |  | | |
|  |  | Yes | 4(3-4) | 16723.0 | .641 |
|  |  | No | 4(3-4) |  |  |
|  | Prior research experience | |  |  |  |
|  |  | Yes | 4(3-4) | 15637.0 | .55 |
|  |  | No | 4(3-4) |  |  |
| AI will have a major impact on healthcare during my career | Gender | |  | | |
|  |  | Male | 4(3-4) | 16171.0 | **<.001** |
|  |  | Female | 4(3-4) |  |  |
|  | Academic level | |  | | |
|  |  | Clinical-year student | 4(3-4) | 17760.0 | **.033** |
|  |  | Pre-residency student | 4(3-4) |  |  |
|  | German language study | |  | | |
|  |  | Yes | 4(3-4) | 16680.0 | **.016** |
|  |  | No | 4(3-4) |  |  |
|  | Personal computer access | |  | | |
|  |  | Yes | 4(3-4) | 16693.0 | .611 |
|  |  | No | 4(3-4) |  |  |
|  | Prior research experience | |  |  |  |
|  |  | Yes | 4(3-4) | 15700.0 | .585 |
|  |  | No | 4(3-4) |  |  |
| I worry that AI will reduce the humanistic aspect of medicine | Gender | |  | | |
|  |  | Male | 4(2.75-4) | 18977.5 | .361 |
|  |  | Female | 4(3-4) |  |  |
|  | Academic level | |  | | |
|  |  | Clinical-year student | 4(3-4) | 19849.0 | .891 |
|  |  | Pre-residency student | 4(3-4) |  |  |
|  | German language study | |  | | |
|  |  | Yes | 4(3-4) | 18460.0 | .516 |
|  |  | No | 4(3-4) |  |  |
|  | Personal computer access | |  | | |
|  |  | Yes | 4(3-4) | 17078.5 | .915 |
|  |  | No | 4(3-4) |  |  |
|  | Prior research experience | |  |  |  |
|  |  | Yes | 4(3-4) | 15208.5 | .309 |
|  |  | No | 4(3-4) |  |  |
| AI will decrease medical errors and misdiagnoses | Gender | |  | | |
|  |  | Male | 3(3-4) | 16931.5 | **.005** |
|  |  | Female | 3(3-4) |  |  |
|  | Academic level | |  | | |
|  |  | Clinical-year student | 3(3-4) | 19655.0 | .752 |
|  |  | Pre-residency student | 3(3-4) |  |  |
|  | German language study | |  | | |
|  |  | Yes | 3(3-4) | 18405.0 | .480 |
|  |  | No | 3(3-4) |  |  |
|  | Personal computer access | |  | | |
|  |  | Yes | 3(3-4) | 15757.5 | .157 |
|  |  | No | 3(3-4) |  |  |
|  | Prior research experience | |  |  |  |
|  |  | Yes | 3(3-4) | 15842.0 | .704 |
|  |  | No | 3(3-4) |  |  |
| I worry that AI will reduce patient trust in physicians | Gender | |  | | |
|  |  | Male | 4(3-4) | 19314.0 | .538 |
|  |  | Female | 4(3-4) |  |  |
|  | Academic level | |  | | |
|  |  | Clinical-year student | 4(3-4) | 18983.0 | .349 |
|  |  | Pre-residency student | 4(3-4) |  |  |
|  | German language study | |  | | |
|  |  | Yes | 4(3-4) | 18679.5 | .652 |
|  |  | No | 4(3-4) |  |  |
|  | Personal computer access | |  | | |
|  |  | Yes | 4(3-4) | 15951.5 | .220 |
|  |  | No | 4(3-4) |  |  |
|  | Prior research experience | |  |  |  |
|  |  | Yes | 4(3-4) | 14072.0 | **.028** |
|  |  | No | 4(3-4) |  |  |
